# Construction and evaluation of a nomogram model for predicting the risk of hospital-acquired pneumonia in elderly patients with acute ischemic stroke

**DOI:** 10.1186/s12877-025-05936-3

**Published:** 2025-05-14

**Authors:** Man Huang, Wan Wang, Wu-lin Li, Yan-qing Chen, Xian-ting Chen, Ye Liu, Yan Li, Dong-mei Ren, Fei Wang

**Affiliations:** 1 Department of Nursing, Jiading District Central Hospital Affiliated Shanghai University of Medicine & Health Sciences, Shanghai, China; 2 Department of Neurology, Jiading District Central Hospital Affiliated Shanghai University of Medicine & Health Sciences, Shanghai, China; 3 Shanghai Key Laboratory of Molecular Imaging, Department of Emergency and Critical Care Medicine, Jiading District Central Hospital Affiliated Shanghai University of Medicine & Health Sciences, Shanghai, China; 4 Department of Emergency and Critical Care Medicine, No.1, Chengbei Rd, Jiading District, Shanghai, China; 5 Department of Nursing, No.111, No.1, Chengbei Rd, Jiading District, Shanghai, China

**Keywords:** Acute ischemic stroke, Hospital-acquired pneumonia, Elderly, Nomogram model

## Abstract

**Objective:**

In this study, we aimed to develop and validate an easy-to-use model to predict the risk of hospital-acquired pneumonia (HAP) in elderly patients with acute ischemic stroke (AIS).

**Methods:**

A total of 2861 elderly AIS patients who were admitted to Jiading District Central Hospital Affiliated with Shanghai University of Medicine & Health Science from January 2016 to December 2023 were selected. Among these patients, 699 were diagnosed with HAP (HAP group), and 2162 patients were included in the control group (non-HAP group). Univariate and multivariate logistic regression analyses were performed to determine the risk factors for HAP after AIS. These factors were then used to establish a scoring system, from which a nomogram model was developed with R software.

**Results:**

Univariate analysis revealed 17 factors that were significantly associated with the development of HAP after AIS in elderly patients (*P* < 0.05). Multivariate logistic regression analysis including these factors revealed that age, the national institute of health stroke scale (NIHSS) score within 24 h of admission (Kwah LK. J Physiother 60:61, 2014), the stress hyperglycemia ratio (SHR), smoking status, and dysphagia status were independent risk factors for HAP after AIS. According to the oxfordshire community stroke project (OCSP) classification, patients classified as having the total anterior circulation infarct (TACI), partial anterior circulation infarct (PACI), and posterior circulation infarct (POCI) sub-types had a significantly increased risk of HAP compared with those classified as having the lacunar infarct (LACI) sub-type. A nomogram model constructed from these six risk factors yielded a C-index of 0.834 (95% confidence interval (CI): 0.811–0.857), indicating high accuracy. Calibration and clinical decision curve analyses revealed the reliability and clinical value of the proposed model.

**Conclusion:**

Our proposed nomogram provides clinicians with a simple and reliable tool for predicting HAP from conventional data. The model can also help clinicians make personalized treatment decisions for patients at different risk levels.

**Clinical trial number:**

Not applicable.

**Supplementary Information:**

The online version contains supplementary material available at 10.1186/s12877-025-05936-3.

## Background

Acute ischemic stroke (AIS) is the necrotic degradation of brain tissue caused by an insufficient cerebral blood supply due to stenosis or occlusion of the cerebral blood supply arteries. AIS accounts for approximately 75–80% of all strokes [1]. It poses a significant health problem for elderly individuals, defined as those aged 65 years and older [2]. The gradual sclerosis of the blood vessel walls and the exacerbation of atherosclerosis with age increase the risk of cerebrovascular diseases [3]. Previous studies indicate that the average age for the onset of ischemic stroke in China is between 65 and 68 years, with men averaging 66.4 years and women 66.6 years [4]. In recent years, China has begun to place greater importance to the adoption of public health strategies and educational measures to raise the awareness of AIS in the elderly population. However, as the elderly population in China grows, the number of patients with AIS also increases [5]. Thus, we should place greater importance on the elderly.

Hospital-acquired pneumonia (HAP) is a common complication among hospitalized elderly stroke patients [6], not only affecting their outcomes but also prolonging the length of hospitalization, increasing their medical burden [7], and even causing death in severe cases. Epidemiological surveys have shown that the incidence of HAP in elderly stroke patients in China is 17–32% [8], whereas that in developed countries is 11.7–14.7% [9–10], indicating that greater awareness and prevention and control strategies for HAP still need to be implemented for elderly stroke patients in China. Previous studies have reported that advanced age, impaired consciousness, difficulty swallowing, and some comorbidities (such as chronic obstructive pulmonary disease (COPD) and atrial fibrillation (AF)) are independent risk factors for the development of HAP in stroke patients [11–14]; notably, impaired nerve reflexes caused by impaired consciousness and swallowing, mucus retention, and reflux aspiration of gastric contents are the main factors influencing the pathogenesis of HAP [15–18]. However, studies assessing the synergistic effects of various risk factors on the risk of HAP after stroke are still lacking.

Nomograms, which are graphical tools built from logistic regression models, can quantify individual disease risk via the integration of the results of risk factor analysis and have been applied to predict the risk of various outcomes of AIS patients [19–20]. Therefore, the purpose of this study was to explore the risk factors for the development of HAP in elderly AIS patients and to establish a predictive nomogram model on the basis of comprehensive clinical data, thereby providing a basis for the individualized prevention and control of HAP in this population.

## Methods

### Study subjects

This investigation was designed as a retrospective study. Data were obtained from an undisclosed internal stroke database of our hospital. AIS patients admitted to Jiading District Central Hospital affiliated with Shanghai University of Medicine & Health Sciences from January, 2016, to December, 2023. All patients included in the analysis met inclusion and exclusion criteria. All patients were treated according to 《 Chinese Guidelines for the Diagnosis and Treatment of Acute ischemic Stroke》 [21] (version 2014 was applied before 2018 and version 2018 was applied after that) after admission.

Diagnostic criteria for AIS [21] include (1) Sudden onset; (2) Presence of focal neurological deficits, such as unilateral weakness or numbness in the face or limbs, language impairments, among others, which may manifest as severe overall neurological dysfunction; (3) Imaging studies revealing definitive lesions or symptoms/signs persisting for over 24 h; (4) Exclusion of non-vascular etiologies; (5) Cerebral CT/MRI conducted to eliminate the possibility of cerebral hemorrhage. The diagnosis must be made by two experienced neurological professionals with at least 5 years of clinical and scientific experience.

HAP [22] refers to infection occurring 48 h after admission to the hospital. The patient presents with cough, cough sputum, audible moist rales in the lungs, fever, elevated white blood cell counts or lung X-rays showing inflammatory infiltrating lesions, or chronic airway diseases such as COPD with new inflammatory infiltrating lesions detected on X-ray after admission to the hospital.

### Inclusion and exclusion criteria

The inclusion criteria were as follows: (1) Individuals diagnosed with AIS through cranial imaging techniques and who fulfill the established diagnostic criteria; (2) The initial cranial computed tomography (CT) scan conducted upon the patient’s admission revealed an absence of cerebral hemorrhage; (3) First onset; (4) age ≥ 65 years [2]; (5) Comprehensive clinical details.

The exclusion criteria were as follows: (1) definite diagnosis of combined community-acquired pneumonia; (2) fever or active infection within 2 weeks prior to admission; (3) prolonged application of immune-modulating pharmaceuticals or glucocorticoids; (4) use of antibiotics prior to admission; (5) a duration exceeding seven days from the onset of symptoms to the point of hospitalization; (6) a recent history that includes notable cranial injuries, episodes of gastrointestinal hemorrhage, and surgical interventions in the abdominal region; (7) incomplete information.

### Data collection

#### The dataset comprises

Demographic information: sex, age, body mass index (BMI, kg/m2) and waist circumference. The data were collected within 24 h after admission.

Previous medical history (diagnostic criteria are shown in Supplementary Table 1): myocardial infarction, hypertensive diseases, diabetes, AF, lipid metabolism disorders, cerebral hemorrhage, dementia, psychiatric disorders, COPD, hemorrhagic disease, family history of stroke, and heart valve replacement surgery. Previous medical history was diagnosed according to the specialist disease diagnostic guidelines. All patients completed history collection and condition assessment, including dysphagia and neurological impairment assessment, within 8 h after admission.

Personal history: smoking status, the oxfordshire community stroke project (OCSP), trial of org 10,172 in acute stroke treatment (TOAST), and symptoms to time of arrival. The data were collected within 24 h after admission.

Severity: per-morbidity modified rankin scale (mRS) score, national institute of health stroke scale (NIHSS) score within 24 h of admission, dysphagia status, mean arterial pressure (MAP), and pulse. Collect within 24 h of admission, the average values of systolic blood pressure (SBP), diastolic blood pressure (DBP) and pulse measured within 24 h of admission were selected respectively, and then the MAP level was calculated and recorded according to the formula: MAP = 1/3 × SBP + 2/3 × DBP.

Treatment: alteplase intravenous thrombolysis, arterial catheter reperfusion therapy, and thrombectomy. Data were collected during hospitalization.

Laboratory parameters: low-density lipoprotein (LDL), homocysteine (Hcy), glycated hemoglobin (HbA1c), admission blood glucose (ABG), stress hyperglycemia ratio (SHR), serum creatinine (SCr), blood urea nitrogen (BUN), uric acid (UA), and the international normalized ratio (INR).completed venous blood collection and laboratory index detection within 24 h after admission.

Clinical prognostic data: HAP status, length of hospitalization, whether death occurred during hospitalization, hospitalization expenses. Extract data from the first page of medical records and discharge bills.

The formula for calculating SHR: SHR = (ABG (mmol/L)/[1.59 × HbA1c (%) − 2.59] [23].

All results are from the first examination after admission to the hospital. The data has been meticulously processed and systematically organized within an Excel database.

### Statistical analysis

Data analysis was performed via R Software (4.2.3). Continuous variables are reported as the means and standard deviations (means ± SDs), M (P25, P75), while categorical variables are reported as percentages, and categorical variables are shown as percentages (%). We compared clinical data across groups via analysis of variance (ANOVA) for continuous variables and either Pearson’s chi-square test or Fisher’s exact test for categorical variables. We use univariate analysis to compare differences between groups. Subsequent to the application of stepwise linear regression to these variables, then multivariate logistic regression analysis was implemented to identify factors associated with HAP. Analyzed the interaction effects of key factors, then modeling the nomogram of HAP after AIS in the elderly using the R package “rms”. In order to evaluate the precision of the nomogram model, a bootstrap approach was implemented, utilizing 1000 resampling iterations to create a plot of the area under the receiver operating characteristic curve (AUROC). To evaluate and contrast the discriminative ability of the nomogram with alternative predictive models, calibration curves will be constructed by plotting the observed probabilities against the predicted probabilities. This approach will facilitate an assessment of the predictive accuracy of the nomogram. Furthermore, we employ decision curve analysis (DCA) to evaluate and contrast the possible net advantages associated with the predictive models. All the statistical analyses were two-tailed, and p values less than 0.05 were considered to indicate statistical significance.

## Results

### Descriptive analysis

The incidence of HAP was significantly higher in patients aged 65 and older, at 24.4%, compared to 7.2% in those under 65 (*P* < 0.001). Furthermore, the study indicated that patients aged 65 and older experienced significantly higher rates of in-hospital mortality (2.1% vs. 0.2%, *P* < 0.001), longer lengths of stay (13 days vs. 11 days *P* < 0.001), and greater total inpatient costs (15.1 thousand yuan vs. 14.1 thousand yuan, *P* < 0.001). For more information, please see Supplementary Table 2.

**Fig. 1:**
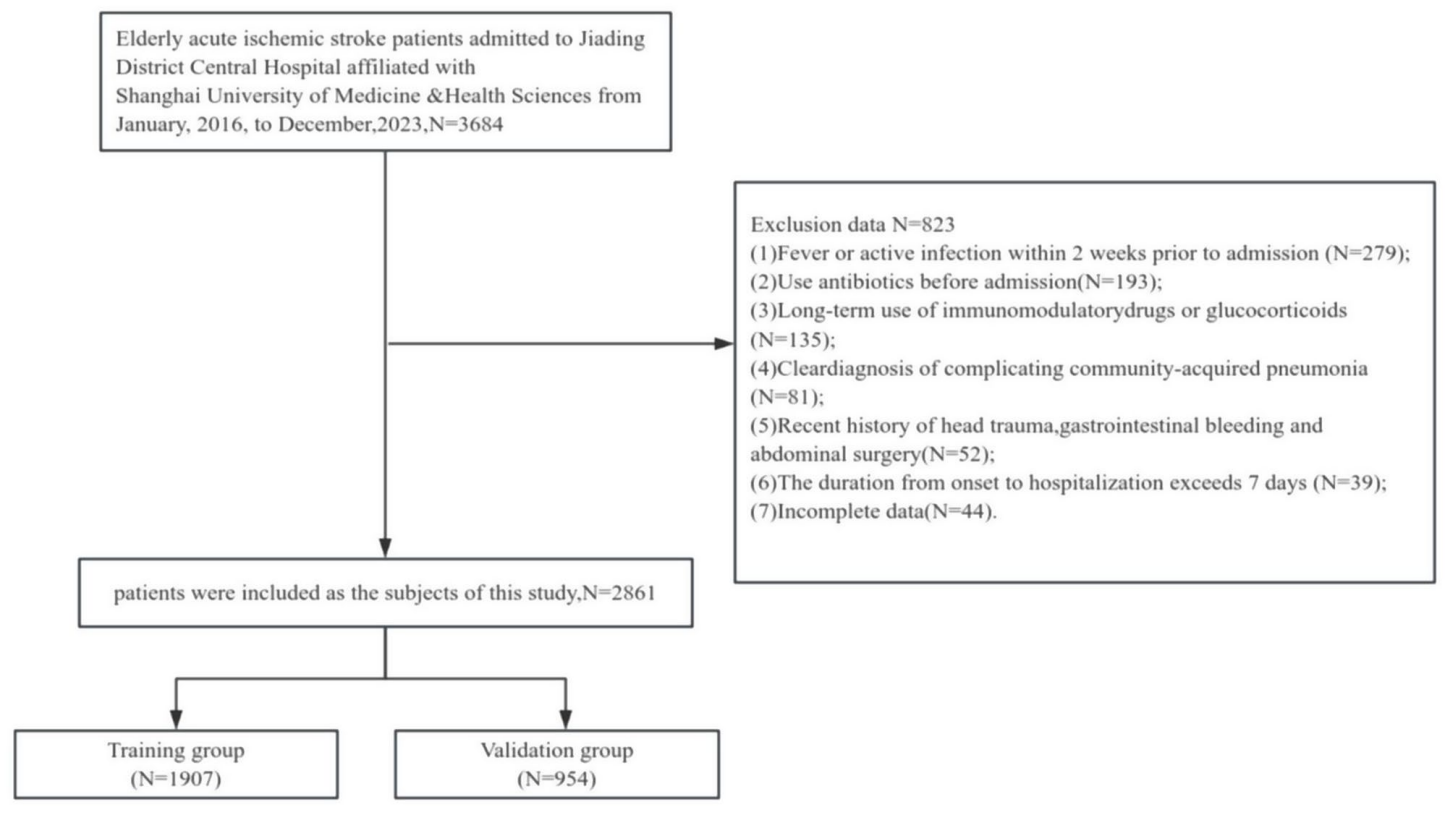
Flow chart

A comprehensive dataset comprising data from 3684 patients was initially used, and 823 patients were subsequently excluded. Ultimately, the study included a total of 2861 patients, with a male preponderance of 57.9% (1656 out of 2861 patients). The average age of the participants was 77.04 years (± 7.84 years). The incidence of HAP was 24.4% (699 out of 2861 patients), accompanied by a mortality rate of 2.1% (61 out of 2861 patients). The participants were divided into two groups on the basis of the occurrence of HAP: the HAP group (*n* = 699) and the non-HAP group (*n* = 2162). The HAP group had a mean age of 80.83 years (± 7.60 years), with males constituting 53.1% (371 out of 699 patients), whereas the non-HAP group had a mean age of 75.81 years (± 7.52 years), with a male preponderance of 59.4% (1285 out of 2162 patients). The patients were randomly assigned to a training cohort (*N* = 1907) or a validation cohort (*n* = 954) at a 2:1 ratio by employing a computerized random number generator. The training cohort was utilized to develop the predictive nomogram model, whereas the validation cohort served to evaluate the performance of the model. Please refer to Fig. 1. and Table 1.

### Comparative analysis of the baseline characteristics between the training and validation groups

The patients were randomly assigned to the training group (*N* = 1907) or the validation group (*N* = 954) at a 2:1 ratio using a computerized random number generator, and the incidence of HAP after AIS did not differ between the two groups. Of the 1907 patients in the training group, 460 were diagnosed with HAP, with an incidence of 24.1%, and of the 954 patients in the validation group, 239 were diagnosed with HAP, with an incidence of 25.1%. There were no significant differences between the two groups in terms of demographic information, previous medical history, personal history, severity, treatment, laboratory parameters, or clinical prognostic data (see supplementary Table 3).

In the overall cohort, the baseline characteristics of the HAP and non-HAP groups were compared, as shown in Table 1.

Baseline data: Compared with the non-HAP group, the HAP group presented statistically significant increases in age and the rates of smoking, TACI sub-type, large-artery atherosclerosis sub-type and cardio embolism sub-type (*P* < 0.05).

Previous medical history: A notable increase in the prevalence of AF was observed in the HAP group relative to the non-HAP group (*P* < 0.05).

Disease severity: Compared with the non-HAP group, the HAP group had a significantly greater mRS score prior to disease onset, NIHSS score recorded at 24 h post admission, incidence of dysphagia, and pulse rate (*P* < 0.05).

Treatment: Compared with the non-HAP group, the HAP group had a significantly greater alteplase intravenous thrombolysis rates and thrombectomy treatment rates (*P* < 0.05).

Laboratory parameters: The Hcy level, ABG values, SHR, SCr level, BUN level, and the INR were significantly elevated in the HAP group compared with the non-HAP group (*P* < 0.05).

Clinical prognostic data: The HAP group had a longer hospitalization duration, higher total hospitalization cost, increased total medication expenses, and an elevated mortality rate compared with the non-HAP group (*P* < 0.05). No significant differences were observed in the remaining indicators between the two groups.

**Table 1:** Baseline characteristics between HAP and non-HAP groups

Variables	Total (*n* = 2861)	Non-HAP (*n* = 2162)	HAP (*n* = 699)	*P*
**Demographic data**				
Male [n (%)]	1656 (57.9)	1285 (59.4)	371 (53.1)	0.003
Age (Mean ± SD, years)	77.04 ± 7.84	75.81 ± 7.52	80.83 ± 7.60	< 0.001
BMI (Mean ± SD)	24.17 ± 2.94	24.19 ± 2.96	24.13 ± 2.88	0.636
Waist circumference (Mean ± SD, cm)	83.35 ± 8.21	83.49 ± 8.22	82.93 ± 8.16	0.136
OCSP [n (%)]				< 0.001
TACI [n (%)]	291 (10.2)	170 (7.9)	121 (17.3)	
PACI [n (%)]	1733 (60.6)	1315 (60.8)	418 (59.8)	
POCI [n (%)]	711 (24.9)	557 (25.8)	154 (22.0)	
LACI [n (%)]	126 (4.4)	120 (5.6)	6 (0.9)	
TOAST [n (%)]				< 0.001
large-artery atherosclerosis [n (%)]	1130 (39.5)	812 (37.6)	318 (45.5)	
Cardio embolism [n (%)]	405 (14.1)	234 (10.8)	171 (24.5)	
small-vessel occlusion [n (%)]	1261 (44.1)	1070 (49.5)	191 (27.3)	
stroke of other determined etiology [n (%)]	23 (0.8)	15 (0.7)	8 (1.1)	
stroke of undetermined etiology [n (%)]	42 (1.5)	31 (1.4)	11 (1.6)	
Symptoms to time of arrival [M (25%,75%), hours]	7.0 (1.9, 28.4)	8.0 (2.1, 31.8)	3.8 (1.4, 17.4)	0.02
Smoking status [n (%)]				< 0.001
Never smoke [n (%)]	2141 (74.8)	1685 (77.9)	456 (65.2)	
Used to smoke [n (%)]	142 (5.0)	107 (4.9)	35 (5.0)	
Still smoke [n (%)]	578 (20.2)	370 (17.1)	208 (29.8)	
**Previous medical history [n (%)]**				
Myocardial infarction [n (%)]	17 (0.6)	10 (0.5)	7 (1.0)	0.107
Hypertensive diseases [n (%)]	2206 (77.1)	1659 (76.7)	547 (78.3)	0.406
Diabetes [n (%)]	870 (30.4)	655 (30.3)	215 (30.8)	0.817
Atrial fibrillation [n (%)]	487 (17.0)	285 (13.2)	202 (28.9)	< 0.001
Lipid metabolism disorders [n (%)]	16 (0.6)	10 (0.5)	6 (0.9)	0.405
Cerebral hemorrhage [n (%)]	71 (2.5)	50 (2.3)	21 (3.0)	0.307
Dementia [n (%)]	40 (1.4)	28 (1.3)	12 (1.7)	0.409
Psychiatric disorders [n (%)]	13 (0.5)	10 (0.5)	3 (0.4)	0.909
Chronic obstructive Pulmonary disease [n (%)]	65 (2.3)	44 (2.0)	21 (3.0)	0.135
Hemorrhagic disease [n (%)]	45 (1.6)	35 (1.6)	10 (1.4)	0.728
Family history of stroke [n (%)]	9 (0.3)	4 (0.2)	5 (0.7)	0.074
Heart valve replacement surgery [n (%)]	4 (0.1)	4 (0.2)	0 (0)	0.578
**The severity of the disease**				
premorbidity mRS score [M (25%, 75%)]	2 (1, 2)	2 (1, 2)	2 (1, 3)	< 0.001
NIHSS score within 24 h of admission [M (25%, 75%)]	2 (1, 7)	2 (1, 4)	8 (2, 15)	< 0.001
Dysphagia [n (%)]	445 (15.6)	106 (5.0)	339 (48.5)	< 0.001
MAP (Mean ± SD, mmHg)	104.32 ± 13.39	104.45 ± 12.92	103.95 ± 14.76	0.391
Pulse (Mean ± SD, Times/minute)	77.64 ± 15.11	76.38 ± 13.76	81.52 ± 18.15	< 0.001
**Special treatment plan**				
Alteplase intravenous thrombolysis [n (%)]	405 (14.2)	289 (13.4)	116 (16.6)	0.033
Arterial catheter reperfusion [n (%)]	22 (0.8)	14 (0.7)	8 (1.2)	0.206
Thrombectomy treatment [n (%)]	39 (1.4)	22 (1.1)	17 (2.5)	0.006
**Laboratory indicators**				
LDL [M (25%,75%), mmol/L]	2.55 (1.94, 3.21)	2.58 (1.97, 3.23)	2.48 (1.83, 3.14)	0.043
Hcy [M (25%,75%), umol/L]	15.5 (11.8, 21.0)	15.0 (11.7, 20.1)	17.7 (12.8, 23.2)	< 0.001
HbA1c (Mean ± SD)	6.61 ± 1.63	6.61 ± 1.62	6.59 ± 1.65	0.665
ABG [M (25%,75%), mmol/L]	5.7 (5.0, 7.2)	5.6 (5.0, 6.9)	6.3 (5.4, 8.0)	< 0.001
SHR (Mean ± SD)	0.85 ± 0.21	0.82 ± 0.18	0.94 ± 0.26	< 0.001
SCr [M (25%,75%), umol/L]	73.50 (62.10, 88.00)	72.84 (62.07, 86.48)	76.44 (62.59, 93.21)	< 0.001
BUN [M (25%,75%), mmol/L]	5.2 (4.3, 6.5)	5.1 (4.2, 6.2)	5.8 (4.6, 7.7)	< 0.001
UA (Mean ± SD, umol/L)	326.61 ± 102.74	325.84 ± 95.90	329.01 ± 121.55	0.478
INR (Mean ± SD)	0.96 ± 0.17	0.95 ± 0.16	1.01 ± 0.19	< 0.001
**Prognostic index**				
Length of hospitalization[M(25%,75%), days]	13 (10, 16)	10 (10, 15)	16 (12, 22)	< 0.001
Died in hospital [n (%)]	61 (2.1)	10 (0.5)	51 (7.3)	< 0.001
Total hospitalization expenses [M (25%,75%), Thousand yuan]	15.0 (11.0, 20.1)	14.2 (11.0, 18.3)	22.0 (14.7, 31.8)	< 0.001
Total hospitalization drug expenses [M (25%,75%), Thousand yuan]	7.2 (4.5, 10.8)	6.8 (4.2, 9.6)	10.8 (6.0, 15.9)	< 0.001
NIHSS score at discharge [M (25%,75%)]	2 (1, 6)	2 (1, 3)	8 (2, 14)	< 0.001
mRS score at discharge [M (25%,75%)]	2 (1, 3)	1 (1, 3)	4 (2, 5)	< 0.001

**Abbreviation**: HAP, hospital-acquired pneumonia; BMI, body mass index; OCSP, oxfordshire community stroke project; TACI, total anterior circulation infarct; PACI, partial anterior circulation infarct; POCI, posterior circulation infarct; LACI, lacunar infarct; TOAST, trial of org 10,172 in acute stroke treatment; mRS, modified rankin scale; NIHSS, national institute of health stroke scale; MAP, mean arterial pressure; LDL, low-density lipoprotein; Hcy, homocysteine; HbA1c, glycated hemoglobin; ABG, admission blood glucose, SHR, stress hyperglycemia ratio; SCr, serum creatinine; BUN, blood urea nitrogen; UA, uric acid; INR, international normalized ratio

### Independent risk factors contributing to the onset of HAP in elderly individuals after AIS

In the training group, the baseline characteristics of the HAP and non-HAP groups were compared, as shown in Supplementary Table 4. To better understand the factors influencing the development of HAP in elderly patients after AIS, a total of 16 factors, including sex, age, OCSP classification, TOAST classification, symptoms to the time of arrival, smoking status, AF status, premorbidity mRS score, NIHSS score within 24 h of admission, dysphagia status, pulse, Hcy level, SHR, SCr level, BUN level, and the INR, were used to establish a linear regression model. Six specific factors—namely, age, the NIHSS score recorded within the first 24 h of admission, the SHR, smoking status, OCSP sub-type, and dysphagia status—were linearly correlated with the incidence of HAP after AIS in the elderly population, as illustrated in Table 2.

**Table 2:** Stepwise linear regression analysis for HAP in elderly AIS patients in the training group

Variables	B	SE	Beta	t	*P*	95%CI
Age	0.01	0.001	0.183	9.081	< 0.001	0.008–0.012
NIHSS score within 24 h of admission	0.01	0.002	0.146	5.688	< 0.001	0.007–0.014
SHR	0.195	0.041	0.095	4.788	< 0.001	0.115–0.275
Smoking status	0.083	0.01	0.154	7.964	< 0.001	0.062–0.103
OCSP sub-type	-0.03	0.012	-0.047	-2.396	0.017	-0.054–0.005
Dysphagia status	0.398	0.03	0.333	13.283	< 0.001	0.399–0.457

**Abbreviation**: NIHSS, national institute of health stroke scale; SHR, stress hyperglycemia ratio; OCSP, oxfordshire community stroke project

Further examination of the six factors was undertaken through multivariate logistic regression analysis. Age, NIHSS score within 24 h of admission and the SHR were used as continuous variables, and categorical variables were used for regression analysis. The findings consistently showed that these six factors serve as independent risk determinants for the occurrence of HAP in elderly patients after AIS. Compared with that of the group with an NIHSS score of 0–4 points, the risk of HAP in the group with an NIHSS score greater than 5 points was significantly greater. Similarly, the risk of HAP was significantly greater in the SHR4 groups than in the SHR1 group, and the risk of HAP was significantly greater in the group older than 80 years than in the group aged 65–80 years, as presented in Table 3.

**Table 3:** Multivariate logistic regression analysis for HAP in the training group

Variables	OR	95%CI	*P*
**Model 1**			
Age	1.084	1.065–1.104	<0.001
NIHSS score within 24 h of admission	1.068	1.04–1.096	<0.001
SHR (continuous)	4.971	2.549–9.692	<0.001
**Smoking status**			
Still smoke	3.697	2.672–5.115	< 0.001
Used to smoke	1.754	0.977–3.152	0.06
Never smoke	Reference		
**OCSP sub-type**			
TACI	7.524	2.035–27.82	0.002
PACI	4.808	1.367–16.914	0.014
POCI	4.677	1.307–16.732	0.018
LACI	Reference		
Dysphagia	6.713	4.552–9.898	< 0.001
**Model 2**			
**Age**			
65–80 years old	Reference		
> 80 years old	2.739	2.087–3.596	< 0.001
**NIHSS score within 24 h of admission**			
0∼4	Reference		
5∼9	1.861	1.322–2.618	< 0.001
10∼14	3.012	1.981–4.579	< 0.001
15∼42	2.964	1.769–4.965	< 0.001
**SHR (quartiles)**			
SHR1 group(0.175–0.726)	Reference		
SHR2 group(0.726–0.820)	1.268	0.857–1.877	0.235
SHR3 group(0.820–0.928)	1.353	0.918–1.992	0.126
SHR4 group(0.928–2.760)	2.371	1.636–3.438	< 0.001
**Smoking status**			
Still smoke	3.263	2.367–4.498	< 0.001
Used to smoke	1.616	0.893–2.922	0.112
Never smoke	Reference		
**OCSP sub-type**			
TACI	7.719	2.059–28.940	0.002
PACI	4.928	1.376–17.644	0.014
POCI	4.955	1.362–18.028	0.015
LACI	Reference		
Dysphagia	7.221	4.911–10.618	< 0.001

The population in this study was grouped according to age [24]: (65–80 years old) and (over 80 years old)

SHR groups the interquartile by value. The value is SHR1 group, SHR2 group, SHR3 group, SHR4 group

NIHSS was divided into 5 groups according to the severity [25], with scores of 0 ~ 4, 5 ~ 9, 10 ~ 14 and 15 ~ 42

**Abbreviation**: NIHSS, national institute of health stroke scale; SHR, stress hyperglycemia ratio; OCSP, oxfordshire community stroke project; TACI, total anterior circulation infarct; PACI, partial anterior circulation infarct; POCI, posterior circulation infarct; LACI, lacunar infarct

### Relationships between OCSP sub-type and the incidence of HAP

Furthermore, the associations between the OCSP sub-type and previously established risk factors, such as NIHSS score within 24 h of admission and dysphagia status were investigated. Compared with patients with LACIs, patients with TACIs, PACIs, and POCIs presented significantly greater incidences of both a higher 24-hour admission NIHSS score and dysphagia (*P* < 0.05) (Table 4).

**Table 4:** Classification of OCSP compared with previously known risk factors in the training group

Variables	Total (*n* = 1907)	TACI (*n* = 190)	PACI (*n* = 1166)	POCI (*n* = 468)	LACI (*n* = 83)	*P*
HAP [n (%)]	460 (24.1)	81 (42.6)	279 (23.9)	96 (20.5)	4 (4.8)	< 0.01
NIHSS score within 24 h of admission [M (25%, 75%)]	2 (1, 7)	6 (2,13)	2 (1,7)	2 (1,4)	1 (0,2)	< 0.01
Dysphagia [n (%)]	287 (15.0)	62 (32.6)	167 (14.3)	56 (12.0)	2 (2.4)	< 0.01

**Abbreviation**: HAP, hospital-acquired pneumonia; NIHSS, national institute of health stroke scale; TACI, total anterior circulation infarct; PACI, partial anterior circulation infarct; POCI, posterior circulation infarct; LACI, lacunar infarct

### Development of a nomogram prediction model

We analysed the interaction effects of the SHR and smoking status, the NIHSS score within 24 h of admission and dysphagia status. Supplementary Fig. 1shows that HAP risk increased with increasing SHR values in patients who never smoked and patients who currently smoked and that the increased rate among patients who currently smoked was greater than that among those who never smoked. Supplementary Fig. 2 shows that with increasing NIHSS scores, the risk of HAP in AIS patients with dysphagia gradually increased, and the increased rate of HAP in patients with dysphagia was greater than that in patients without dysphagia.

**Fig. 2:**
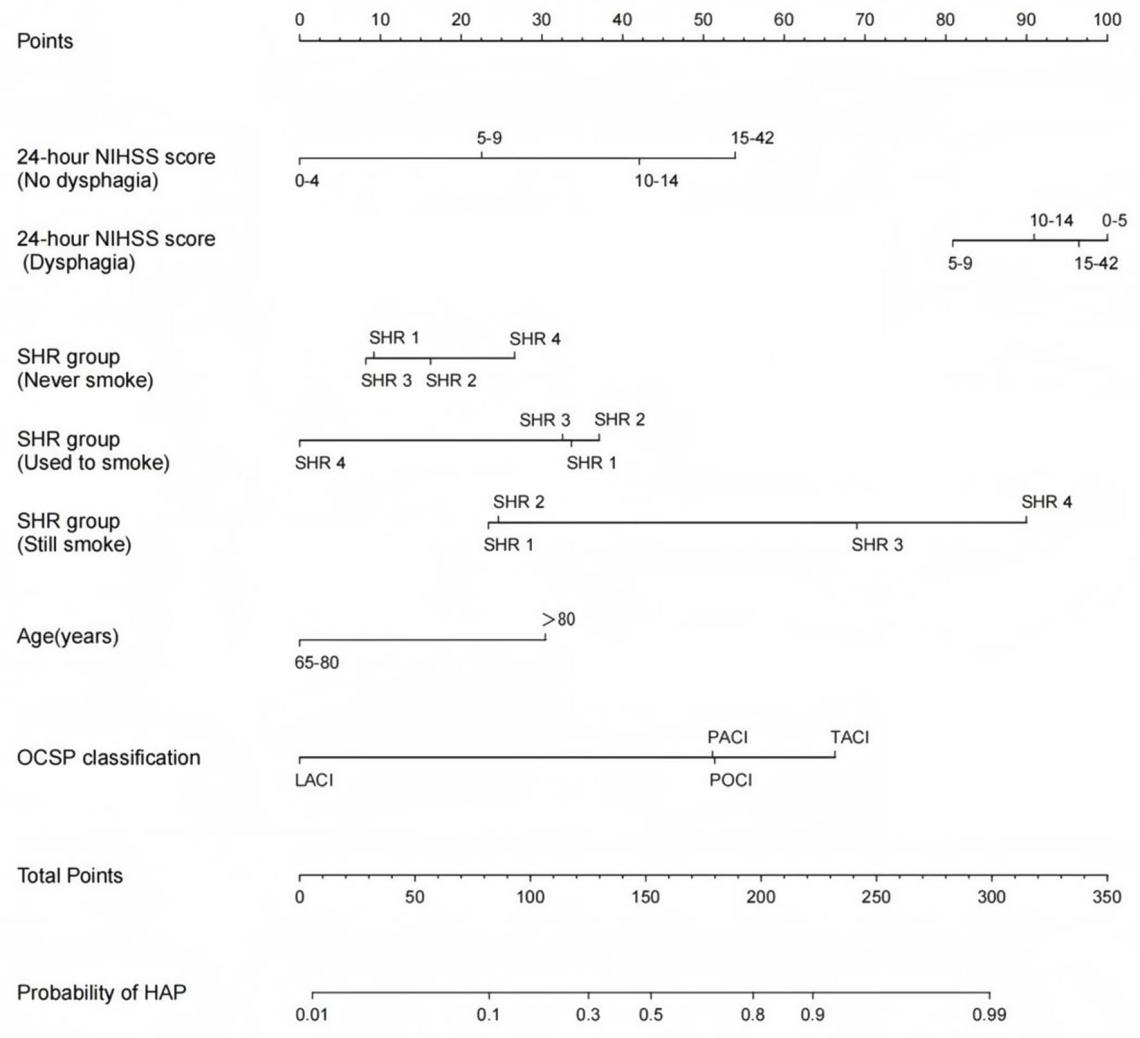
A nomogram model to predict HAP risk in elderly patients with AIS

We also developed HAP prediction graphs with R software using six different independent risk factors, including age, NIHSS score within 24 h of admission, SHR value, smoking status, OCSP sub-type, and dysphagia status. Analysis showed significant interactions: one between the NIHSS score and dysphagia status (*P* < 0.001), and another between SHR values and smoking status (*P* < 0.001). The term “points”, which is displayed at the top of the model, reflects the score associated with each risk factor. This model was designed to compute individual scores for each independent risk predictor, ultimately yielding a “total points.” The “probability of HAP” value located in the lower section of the model corresponds to the “Total Points” and represents the estimated probability of HAP in elderly patients after AIS, as illustrated in Fig. 2.

### The predictive ability of the nomogram model

**Fig. 3:**
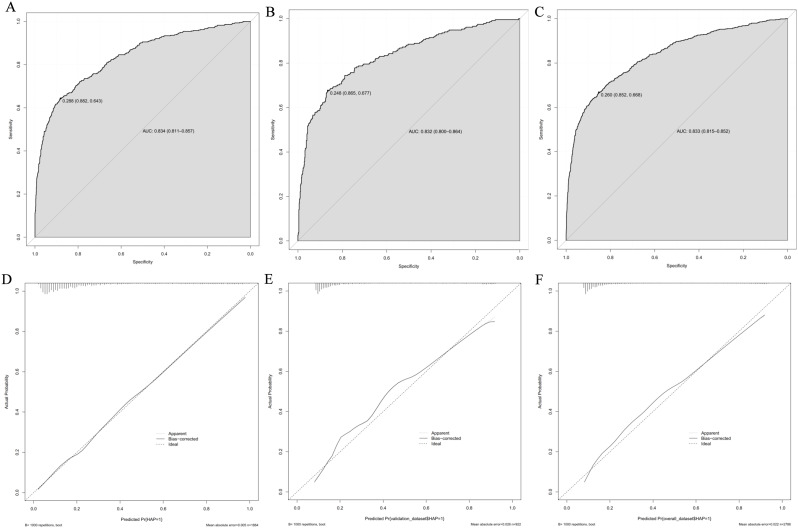
**A**. Comparison of area under the receiver operating characteristic curve (AUROC) values among different scoring systems for prediction of HAP, in the training group. **B**. Comparison of area under the receiver operating characteristic curve (AUROC) values among different scoring systems for prediction of HAP, in the Validation group. **C**. Comparison of area under the receiver operating characteristic curve (AUROC) values among different scoring systems for prediction of HAP, in the overall cohort. **D**. Calibration curve of the nomogram for the training group. **E**. Calibration curve of the nomogram for the Validation group. **F**. Calibration curve of the nomogram for the overall cohort

The ROC analysis revealed that the area under the curve (AUC) values for the nomogram model were 0.834 (95% confidence interval [CI]: 0.811–0.857) in the training cohort, 0.832 (95% CI: 0.800–0.864) in the validation cohort, and 0.833 (95% CI: 0.815–0.852) in the overall cohort (refer to Fig. 3A and B, and 3C). These findings suggest that the model has robust discriminative ability. Furthermore, the estimated risk of HAP in elderly patients after AIS strongly agreed with the actual risk, as evidenced by the mean absolute error of 0.005 in the training cohort (illustrated in Fig. 3D). Additionally, the calibration plots comparing the predicted risk of HAP against the actual risk in both the validation cohort and the overall cohort further supported this consistency, with mean absolute errors of 0.026 and 0.022, respectively (depicted in Fig. 3E and F). These results collectively indicate that the nomogram prediction model possesses a high degree of accuracy.

### Assessment of the nomogram developed for clinical Decision-Making

**Fig. 4:**
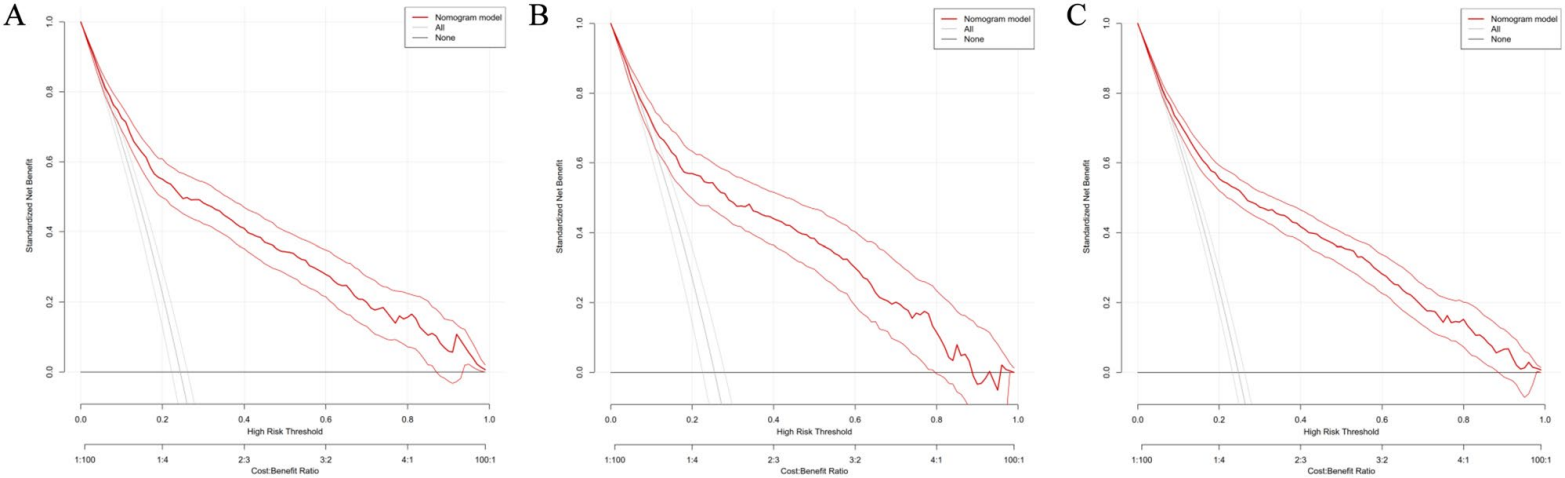
**A**. Clinical impact curves of nomogram model in the training group. **B**. Clinical impact curves of nomogram model in the validation group. **C**. Clinical impact curves of nomogram model in the overall cohort

In this study, DCA was conducted using the high-risk threshold probability represented on the horizontal axis and the net benefit rate illustrated on the vertical axis. The range for the high-risk threshold probability was established between 0 and 1. The resulting DCA curves revealed that the nomogram model could offer a net clinical benefit for patients in the training cohort (refer to Fig. 4A), the validation cohort (see Fig. 4B), and the overall cohort (illustrated in Fig. 4C) across all threshold probabilities ranging from 0.01 to 0.99.

## Discussion

This study revealed that the incidence of HAP after AIS is relatively high in elderly patients and that these patients have higher mortality rates, total hospitalization costs, and drug consumption costs and longer hospitalization stays than elderly patients without HAP after AIS do. In this study, a nomogram model was developed and validated to predict the development of HAP in elderly patients after AIS to aid in the prevention and treatment of this disease.

Research showed that elderly patients have a higher incidence of HAP following AIS [26–29]. This rate is notably higher than that of non-elderly individuals, which is approximately 11.7–14.7% [9–10]. The results of this study reinforce these findings, indicating a rate of 24.4% in elderly patients compared to only 7.2% in younger patients. To this end, we employed a difference test and multivariate logistic regression analysis to identify independent risk factors for the development of HAP in elderly patients after AIS. A nomogram was then constructed from these independent risk factors, which included age, the NIHSS score within 24 h of admission, the SHR, smoking status, and the OCSP classification and dysphagia status. The nomogram showed good classification and calibration abilities in the training cohort and validation cohort, suggesting that it can be used as a simple and convenient tool in clinical practice to score elderly patients’ probability of developing HAP after AIS. In addition, weighing the benefits of individualized clinical decision-making can help clinicians determine risk management strategies for elderly stroke patients. The possible reasons by which these factors affect HAP are as follows:

(1) Age: Some studies have shown that the fatality rate of pneumonia increases with increasing age, from 74.7 cases/100,000 people over 65 years of age to 77.2 cases/100,000 people aged 75 ~ 84 years and 294.7 cases/100,000 people aged 85 years and older [30]. Two large cohort studies have shown that AIS patients older than 65 years have an elevated risk of developing pneumonia. Additionally, the incidence of pneumonia in elderly males and in patients with high NIHSS scores was 1–2 times greater than that in younger patients and those with low NIHSS scores, respectively [31]. Moreover, among elderly AIS patients, HAP patients are significantly older than non-HAP patients are [32], which may be related to the fact that older people have more comorbidities and impaired swallowing disorders [33].

(2) NIHSS score: The NIHSS score is widely used in clinical practice to assess the severity of stroke; a higher score indicates potentially greater brain tissue damage, neurological dysfunction, or bulbar paralysis, which may affect a patient’s respiratory function, causing difficulty in discharging respiratory secretions and thus increasing the risk of HAP development [34, 35]. In addition to reflecting the severity of neurological impairment, the NIHSS score is also an independent risk factor for the development of stroke-associated pneumonia (SAP) [36, 37]. In particular, when the NIHSS score is ≥ 5 points, the risk of SAP is significantly increased [38]. In AIS patients, a high NIHSS score is an independent risk factor for the development of HAP. In addition, the NIHSS score has been closely associated with swallowing disorders [39]. A higher score indicates a lower degree of neurofunctional area and neural network structure reorganization and a more difficult recovery from swallowing disorders [40]. Greater attention should be given to patients with high NIHSS scores, and preventive measures for controlling the risk factors for HAP should be implemented.

(3) Previous studies have reported a definite correlation between the SHR and the outcomes of patients with ischemic stroke; moreover, the SHR is strongly influenced by basal blood glucose levels. The SHR has been confirmed to be superior to ABG analysis in predicting the development of HAP in AIS patients [41]. This may be related to the following mechanisms. Hyperglycaemia reduces vascular endothelial nitric oxide levels and promotes vasoconstriction, which leads to abnormal organ perfusion [42]. Hyperglycaemia also leads to oxidative stress, promotes the combinatory reactions of cytokines, and aids in the development of excessive inflammatory responses, exhausting the immune system while directly inhibiting immunoglobulin, lymphocytes and the complement system [43]. These factors may eventually lead to systemic immune function suppression, thus increasing susceptibility to stroke-induced immunosuppression syndrome and poststroke infection in elderly patients [44]. Finally, hyperglycaemic patients who have previously experienced metabolic disorders and immune dysfunction are more likely to have difficulty swallowing and delayed oesophageal emptying, which subsequently increases the risk of HAP [45].

(4) Smoking status is significantly associated with the development of HAP in elderly patients after AIS. Cigarettes contain a variety of harmful substances; consequently, long-term smoking can damage airway function and lung tissues and decrease immune function [46]. Furthermore, smoking damages airway cilia, reducing their ability to remove mucus and pathogens in the lungs and thus leading to pathogen accumulation. Smoking can also significantly reduce the mucociliary transport velocity (from 0.14 ± 0.03 mm/s to 0.00 mm/s) [47], while smoking-induced inflammation and oxidative stress damage the integrity of the airway epithelium and weaken the defence mechanisms against infection [48]. This further increases the possibility of the development of HAP in AIS patients who smoke.

(5) Difficulty swallowing is a common comorbidity of pneumonia after stroke, with a prevalence as high as 45% [49]. This study revealed that 48.5% of HAP patients experienced difficulty swallowing, supporting the findings of previous studies. Patients with dysphagia also tend to present dysfunction of the chewing and swallowing muscles [50], which makes it difficult for them to take in nutrients and increases the risk of hypoalbuminaemia and immune weakening, thereby increasing the risk of SAP. Furthermore, swallowing dysfunction is closely related to aspiration in patients after stroke [19]. Approximately 37–78% of patients with acute stroke will develop swallowing disorders, among whom approximately half will develop aspiration; the risk of developing pneumonia increases when aspirates contain food residues, digestive juices or oropharyngeal secretions [51]. In addition, unilateral basal ganglia infarction can affect the normal metabolism of dopamine, reducing the secretion of substance P by the vagus and swallowing nerves to a certain extent, thereby causing swallowing disorders and further weakening of the cough reflex and ultimately leading to the development of SAP [52].

(6) This study revealed that in the OCSP classification of elderly AIS patients, compared with that of patients with the LACI sub-type, the risk of HAP in patients with the other sub-types was significantly increased, which further supported the findings of Li SJ, Teh WH et al. [9, 53]. Possible causes include the following: (1) The TACI sub-type often involves the main middle cerebral artery and manifests as severe hemiplegia, hemisensory disorder and functional dysfunction of the higher cortex, bulbar paralysis and decreased airway protection ability, thus increasing the risk of HAP [54], and some TACI patients have decreased consciousness, resulting in a weakened cough reflex and difficulty in sputum expulsion. This further increases the risk of pneumonia [55]. (2) PACI, which involves the middle cerebral artery branches, affects the ability of autonomous sputum excretion and extends the time spent in bed, leading to an increased risk of HAP [56]. (3) POCI, which mainly involves the medulla oblongata (such as the nucleus tractus solitatus and nucleus hypochtholus), can directly damage the cough reflex arc [57], resulting in decreased airway secretion clearance ability and thus increasing the risk of HAP [58].

(7) Previous research indicates that AF is a risk factor for pneumonia [59, 60]. However, this study’s findings do not align with previous research for several reasons: (1) The study population was inconsistent. This study focuses on individuals aged 65 and older, while Yuan et al. [59]. reported that 57.2% of their participants were in the same age group, which may explain the differing results. (2) The baseline characteristics of the study population were inconsistent. In comparison to Yuan et al.‘s findings, this study found no significant difference in the proportion of AF among the total population [17% (487/2861) vs. 18.8% (85/451), *P* = 0.341]. However, the proportion of AF in patients with pneumonia was significantly lower than reported by Yuan et al. [28.9% (202/699) vs. 45.9% (45/98), *P* = 0.001]. (3) The outcomes of studies were inconsistent. Curro CT’s [60] study focused on the relationship between asymptomatic pulmonary opacities and AF, whereas this study specifically investigated HAP, which includes but is not limited to patients with APO. This difference may also contribute to the inconsistent results.

On the basis of these 6 independent risk factors, we created a model to predict HAP in elderly patients after AIS and then presented the model as a nomogram. In the training group, the validation group, and the overall cohort, the AUROCs were 0.834 (95% confidence interval (CI): 0.811–0.857), 0.832 (95% CI: 0.800–0.864), and 0.833 (95% CI: 0.815–0.852), respectively, indicating that the nomogram model has good predictive value. This potentially makes the model suitable for helping identify high-risk patients and providing timely support and anti-infection treatment to minimize the incidence of poor outcomes in HAP patients. The nomogram can visually reflect the quantitative score of each risk factor and be used to calculate the predictive value and corresponding risk level of individual outcome events by integrating the cumulative risk values for each factor.

Our study has several significant advantages. First, this was a retrospective cohort study in which a validated nomogram tool was developed to predict HAP in elderly patients after AIS; few such studies have been conducted previously. Second, our study included a relatively large clinical cohort, which helped increase the strength of the evidence and allowed for the acquisition of large amounts of information on the factors that influence the development of HAP in elderly patients after AIS. We also explored the incidence of HAP as a function of the OCSP classification in AIS patients, again providing evidence for clinicians to attach importance to this classification.

However, despite these advantages, we must also note some limitations of this study. First, this was a single-centre study; assessment of the nomogram with multicentre data could increase the validity of the model. The retrospective design and reliance on data from a single centre also introduced inherent biases that may have affected the validity of our results. To overcome these limitations in future studies, we plan to use data from multiple centres in China to improve the generalizability of our results. Second, our analysis of the risk factors was not comprehensive; information on indicators, including C-reactive protein, neutrophil percentage, and mechanical ventilation status, was not collected in this study. Our data were obtained from an undisclosed internal stroke database of the hospital, which does not include data related to these indicators. In future studies, we will ensure more scientific and comprehensive data collection. Third, this study found that the prevalence of dementia was 1.4%, notably lower than the 5.5% prevalence reported in China for individuals over 60 years old (3.9% for Alzheimer’s disease, 1.6% for vascular dementia) [61]. The low prevalence may be attributed to several factors: 1. Patients or guardians, influenced by traditional customs, hide symptoms or avoid seeking medical treatment due to fear of social discrimination or psychological pressure. 2.Clinicians gathered the present illness history, past medical history, and assessed the patient’s condition upon admission. Our hospital is a general medical facility that does not serve as a psychiatric institution. A dementia diagnosis usually requires the medical history provided by the patient’s guardian. The patient might not have sought medical attention for past dementia-related symptoms, leaving the guardian unaware of a potential dementia diagnosis. As a result, this may lead to missed opportunities for diagnosing dementia. 3. Some patients showed signs of aphasia when admitted, and their guardians frequently failed to mention any previous dementia diagnosis. Some guardians even mistake early symptoms, such as forgetfulness and personality changes, for normal aging rather than disease. This lack of information poses a significant challenge for clinicians, potentially leading to the underdiagnosis of dementia. These reasons may be responsible for the low prevalence of dementia. Due to the underdiagnosis of dementia, its impact as a risk factor for HAP may have been underestimated in this study.

## Conclusion

On the basis of routinely collected data, we created and validated a reliable nomogram for predicting the individualized risk of pneumonia after stroke that showed good discrimination ability and accuracy. The proposed nomogram could serve as a simple and useful tool for clinicians to make timely individualized clinical decisions on the basis of the risk predicted for individual patients.

## Electronic supplementary material

Below is the link to the electronic supplementary material.

Supplementary Material 1

Supplementary Material 2

Supplementary Material 3

Supplementary Material 4

Supplementary Material 5

Supplementary Material 6

## Data Availability

The data that support the findings of this study are available from an undisclosed internal stroke database of our hospital. The database was established at the end of 2013 on the basis of the “Shanghai Stroke and Treatment Service System” and the quality control requirements of the neurology department. Access to these data is restricted, as they were utilized under license for the present study and are therefore not publicly accessible. Nevertheless, the data are available from the corresponding author upon reasonable request.
