# Supplementary figures and images for: Construction and evaluation of a nomogram model for predicting the risk of hospital-acquired pneumonia in elderly patients with acute ischemic stroke
Source: BMC Geriatr. 2025 May 14;25:340. doi: 10.1186/s12877-025-05936-3 (PMC12080133; doi:10.1186/s12877-025-05936-3)

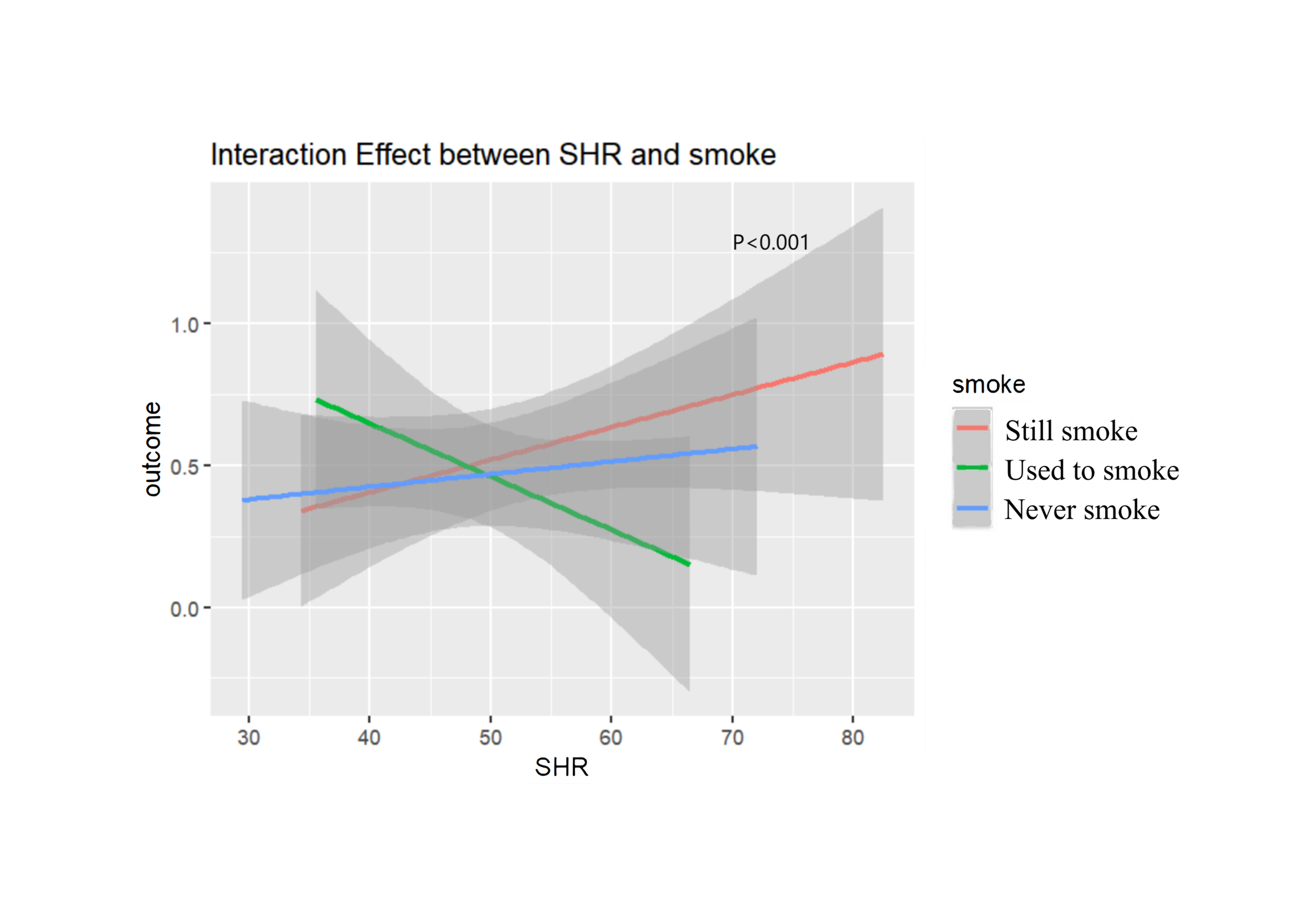

Supplement: Supplementary file 1 — Supplementary Material 1 [file 12877_2025_5936_MOESM1_ESM.tif]

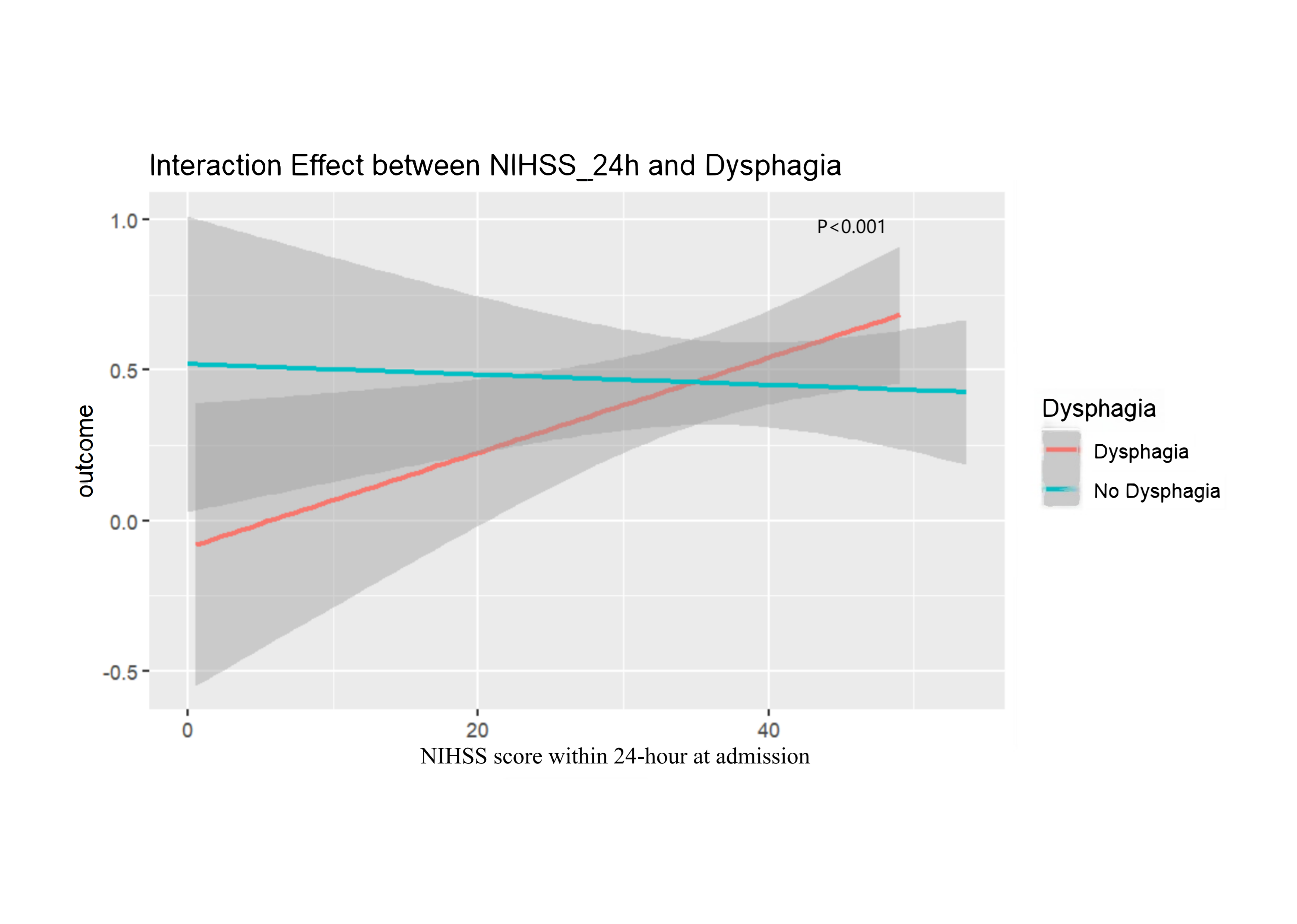

Supplement: Supplementary file 2 — Supplementary Material 2 [file 12877_2025_5936_MOESM2_ESM.tif]
