# Supplementary material for: Construction and evaluation of a nomogram model for predicting the risk of hospital-acquired pneumonia in elderly patients with acute ischemic stroke
Source: BMC Geriatr. 2025 May 14;25:340. doi: 10.1186/s12877-025-05936-3 (PMC12080133; doi:10.1186/s12877-025-05936-3)
Supplement: Supplementary file 3 — Supplementary Material 3 [file 12877_2025_5936_MOESM3_ESM.docx]

**Supplementary table 1 Diagnostic Criteria for Medical History**

**1) Diagnostic criteria for myocardial infarction:** 2018 and before reference, “Third universal definition of myocardial infarction” ; for 2019 and beyond reference, “Fourth Universal Definition of Myocardial Infarction (2018)” .

The specific diagnostic criteria are as follows: an increase and/or decrease in cTn is detected, and at least one value is above the upper limit of the 99th percentile reference value (URL) and meets at least one of the following clinical criteria for myocardial ischaemia: ① symptoms of acute myocardial ischaemia; ② new ischaemic electrocardiogram changes; ③ development of a pathological Q-wave; and (4) imaging evidence of the loss of new viable myocardia or new local wall motion abnormalities; coronary angiography or autopsy.

1. **The diagnostic criteria for hypertension** in 2018 and before were based on the Chinese Guidelines for Hypertension Prevention and Treatment (2010) . The diagnostic criteria are as follows:

① In the absence of antihypertensive drug use, a systolic blood pressure ≥140 mmHg and/or a diastolic blood pressure ≥90 mmHg are further classified into grades 1, 2, and 3 hypertension according to elevated blood pressure. It is generally necessary to perform three measurements on different days to determine the blood pressure and its classification, especially for mild and moderate hypertension.

② Cardiovascular risk stratification: Patients were divided into low-risk, medium-risk, high-risk and very high-risk 4 groups according to the blood pressure, cardiovascular risk factors, target organ damage, clinical complications and diabetes status.

③ Grade 3 hypertension with one or more risk factors combined with diabetes mellitus; patients with complications such as clinical heart disease, cerebrovascular disease or chronic kidney disease are at high cardiovascular risk.

In 2019 and beyond, according to the Chinese Guidelines for Hypertension Prevention and Treatment (2018) , the diagnostic criteria are as follows:

① In the absence of antihypertensive drug use, a blood pressure in the consulting room ≥140/90 mmHg, a family blood pressure ≥135/85 mmHg, an 24-hour ambulatory blood pressure ≥130/80 mmHg, a daytime blood pressure ≥135/85 mmHg, and a night blood pressure ≥120/70 mmHg.

② Hypertension was divided into grades 1, 2, and 3 according to the elevated blood pressure level in the consulting room (1, C).

③ Cardiovascular stratification was performed according to blood pressure, cardiovascular factors, target organ damage, clinical complications, and complications such as diabetes and CKD, which were divided into four levels: low risk, medium risk, high risk and very high risk (1, C).

**3) Diagnostic criteria for diabetes:** 2018 and before reference“Management of hyperglycemia in type 2 diabetes, 2015”; for 2019 and beyond reference “Management of Hyperglycemia in Type 2 Diabetes, 2018”.

The specific diagnostic criteria are associated with diabetes symptoms (such as polydipsia, polyuria, polydipsia, and unexplained weight loss), and one of the following conditions must be met:

① A fasting blood glucose ≥7.0 mmol/L.

② An oral glucose tolerance test (OGTT) 2-hour blood glucose level ≥ 11.1 mmol/L.

③ A haemoglobin a1C (HbA1c) level ≥6.5%.

④ A random blood glucose level ≥ 11.1 mmol/L.

**4) Diagnostic criteria for atrial fibrillation**: Before 2021 reference“2014 AHA/ACC/HRS guideline for the management of patients with atrial fibrillation” , for 2022 and beyond reference“Management of Atrial Fibrillation in 2021”. The specific diagnostic criteria are as follows:

① Absolute arrhythmia: irregular R‒R interval (no repetitive pattern).

② P wave disappearance: There is no clear regular P wave generated by atrial depolarization at ECG baseline.

③ Atrial fibrillation waves: These waves are replaced by fast, disordered low-amplitude fibrillation waves ( f waves, usually 350–600 times/min).

1. **Diagnostic basis of lipid metabolism** disorders “The Diagnosis and Management of Lipodystrophy Syndromes” . The specific diagnostic criteria are as Table 1.

Table 1 : Chinese ASCVD Primary Prevention Population Lipid Suitable Levels and Abnormal Stratification Standards [mmol/L (mg/dl)]

| Stratification | TC | LDL-C | HDL-C | Non-HDL-C | TG |
| --- | --- | --- | --- | --- | --- |
| Ideal Level | - | <2.6(100) | - | <3.4(130) | - |
| Suitable Level | < 5.2(200) | <3.4(130) | - | <4.1(160) | <1.7(150) |
| Borderline Elevated | ≥ 5.2(200)and <6.2(240) | ≥3.4(130)and<4.1(160) | - | ≥4.1(160)and<4.9(190) | ≥1.7(150)and<2.3(200) |
| Elevated | > 6.2(240) | ≥4.1(160) | - | ≥4.9(190) | ≥2.3(200) |
| Decreased | - | - | <1.0(40) | - | - |

1. **Diagnostic criteria for cerebral haemorrhage**: Before 2019 Reference, “Chinese guidelines for diagnosis and treatment of acute intracerebral hemorrhage 2014; for 2020 and beyond reference, Chinese guidelines for the diagnosis and treatment of acute intracerebral hemorrhage 2019” . The specific diagnostic criteria are as follows:

① Acute onset;

② Symptoms of functional impairment of the Vesta channel (a few are comprehensive neurological impairments), often accompanied by headache, vomiting, elevated blood pressure and varying degrees of consciousness disorders;

③ Head CT or MRI showing a bleeding focus;

④ Nonvascular cerebral causes are excluded.

7) The mental diseases mentioned in this database refer to primary psychiatric disorders, including schizophrenia, psychoaffective disorders, schizoaffective disorders, delusional disorders, compulsive and related disorders and dissociative disorders. Since our hospital is a nonpsychiatric hospital, the collection of data for this item in this database relies on the medical history of the psychiatric hospital provided by the patient's guardian.

8)**Diagnostic criteria for chronic obstructive pulmonary disease**: before 2021 “Guidelines for Diagnosis and treatment of chronic obstructive pulmonary disease (2013 Revised edition)” ，2022 and beyond basis “Primary Diagnosis and Treatment Guide for chronic obstructive pulmonary Disease” . The diagnostic criteria are as follows:

① Signs and symptoms (must meet at least one):

Persistent dyspnoea: Progressive aggravation (noticeable after activity, which may occur at rest as the disease progresses).

Chronic cough: Recurrent cough with or without sputum (may be intermittent, but other causes should be ruled out).

Risk factor exposure history: long-term smoking (≥10 pack-years), biofuel smoke exposure, occupational dust/chemical exposure, etc.

② Pulmonary function test (necessary for diagnosis):

An FEV/FVC after bronchodilator use < 0.70 indicates persistent airflow limitation (not completely reversed).

Classification and severity (based on the FEV₁ percentage of the estimated value):

GOLD 1 (mild): FEV₁ ≥80%

GOLD 2 (medium): 50% ≤FEV₁ <80%

GOLD 3 (heavy): 30% ≤FEV₁ <50%

GOLD 4 (very heavy): FEV₁ amount <30%

| 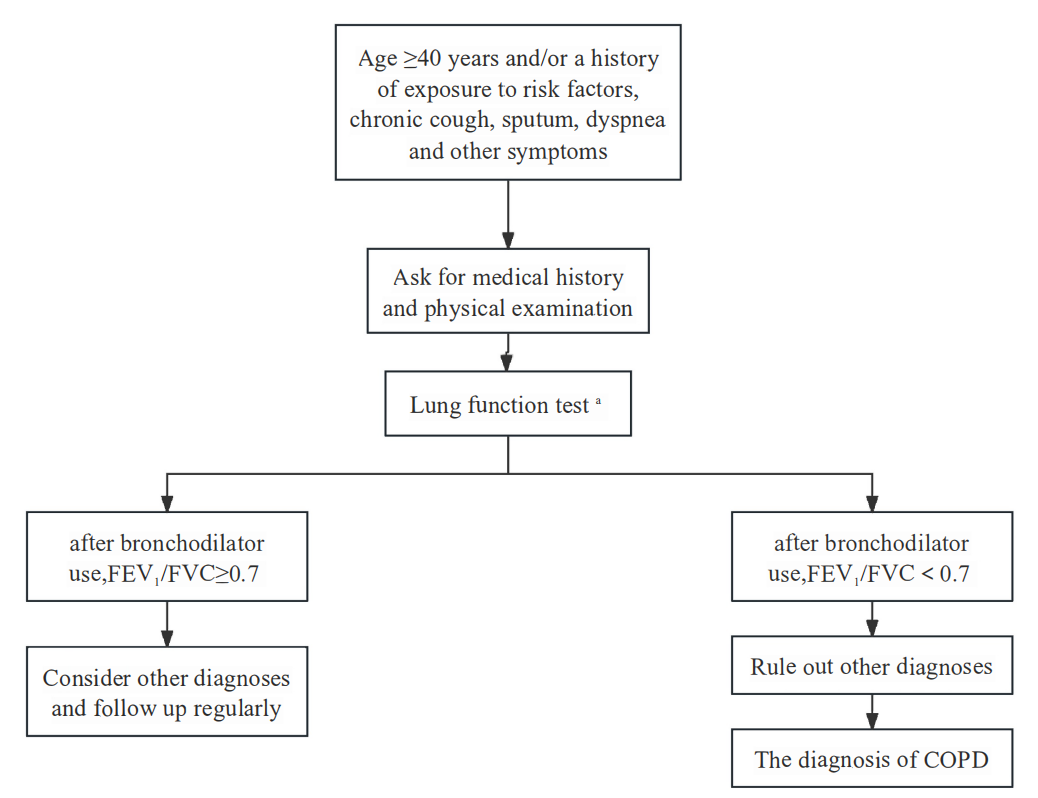 |
| --- |
| Note:^a^When the primary hospital does not have the conditions for pulmonary function examination, the high-risk individuals with COPD can be found through screening questionnaires, and the suspected patients should be referred to higher hospitals for further diagnosis. Regular follow-up is recommended for non-high-risk individuals.  Figure Diagnosis and treatment flow of chronic obstructive pulmonary disease (COPD)  *Note:^a^When the primary hospital does not have the conditions for pulmonary function examination, the high-risk individuals with COPD can be found through screening questionnaires, and the suspected patients should be referred to higher hospitals for further diagnosis. Regular follow-up is recommended for non-high-risk individuals.*  *Figure 5 Diagnosis and treatment flow of chronic obstructive pulmonary disease (COPD)* |

1. **Haemorrhagic diseases** refer to diseases characterized by spontaneous bleeding or persistent posttraumatic bleeding due to abnormal haemostasis mechanisms (including blood vessel walls, platelets, coagulation factors, fibrinolytic systems and anticoagulant substances). The collection of data for this item in this database relies on the medical history provided by the patient or their guardian and a clear diagnosis.
2. **Dementia** primarily includes Alzheimer's disease and vascular cognitive impairment, based on the medical histories provided by their caregivers.

10.1 The main diagnostic criteria for Alzheimer's disease include: (1) an insidious onset with progressive deterioration affecting work and daily functioning; (2) cognitive impairment primarily characterized by memory loss, accompanied by gradual decline in areas such as language, visuospatial skills, and executive function; and (3) noticeable changes in personality, mental activity, and behavior.

10.2 Key diagnostic points for vascular cognitive impairment are as follows:

1. Cognitive function assessments identify the presence of cognitive impairment, which may consist of vascular dementia, VaMCI, or focal cortical dysfunction.

2.Cerebrovascular diseases are associated with cognitive impairment, typically manifesting suddenly. Cognitive impairment usually begins after one or more cerebrovascular events. The disease may progress in an inconsistent pattern, either after multiple cerebrovascular events or without a stroke or transient ischemic attack. The condition has a gradual onset and slow progression, which can be measured by assessing information processing speed, complex attention, and significant impairments in the executive functions of the frontal lobe, along with one of the following characteristics: (1) Early gait abnormalities; (2) Increased urinary frequency and urgency, or other urinary symptoms not linked to the urinary system or neurological conditions; (3) Changes in personality or other signs of subcortical impairment.

3. Brain imaging provides evidence that links cerebrovascular disease to cognitive impairment.

A diagnosis of vascular cognitive impairment (probable VCI) is likely if both condition 2 and condition 3 are satisfied. If condition 2 is met without an imaging examination, or if the imaging results do not fully clarify the cognitive impairment, we diagnose possible vascular cognitive impairment ( possible VCI ). The absence of abnormalities in imaging is a critical criterion for ruling out a diagnosis of vascular cognitive impairment.
