# Supplementary material for: Construction and evaluation of a nomogram model for predicting the risk of hospital-acquired pneumonia in elderly patients with acute ischemic stroke
Source: BMC Geriatr. 2025 May 14;25:340. doi: 10.1186/s12877-025-05936-3 (PMC12080133; doi:10.1186/s12877-025-05936-3)
Supplement: Supplementary file 4 — Supplementary Material 4 [file 12877_2025_5936_MOESM4_ESM.doc]

Supplementary Table 2 Comparison of prognostic data for IS patients aged 65 and older versus those under 65

| Variables | Total  (n = 4330) | ＜65 years  (n=1469) | ≥65 years  (n=2861) | *P* |
| --- | --- | --- | --- | --- |
| HAP [n (%)] | 804 (18.6) | 105 (7.2) | 699 (24.4) | <0.001 |
| Died in hospital [n (%)] | 64 (1.5) | 3 (0.2) | 61 (2.1) | <0.001 |
| Length of hospitalization[M(25%,75%), days] | 12.00 (10, 15) | 11.00 (10, 14) | 13.00 (10, 16) | <0.001 |
| Total hospitalization expenses [M (25%,75%), Thousand yuan] | 14.7 (11.3, 2.0) | 14.1 (11.1, 18.4) | 15.1 (11.5, 20.7) | <0.001 |
| Total hospitalization drug expenses [M (25%,75%), Thousand yuan] | 7.1 (4.5, 10.4) | 6.8 (4.3, 9.8) | 7.2 (4.5, 10.9) | <0.001 |

**Abbreviation:** HAP, hospital-acquired pneumonia.
