# Supplementary material for: Construction and evaluation of a nomogram model for predicting the risk of hospital-acquired pneumonia in elderly patients with acute ischemic stroke
Source: BMC Geriatr. 2025 May 14;25:340. doi: 10.1186/s12877-025-05936-3 (PMC12080133; doi:10.1186/s12877-025-05936-3)
Supplement: Supplementary file 5 — Supplementary Material 5 [file 12877_2025_5936_MOESM5_ESM.doc]

Supplementary table 3 Baseline characteristics of the training and validation cohorts

| Variables | Total (n = 2861) | Training group (n = 1907) | Validation group (n = 954) | *P* |
| --- | --- | --- | --- | --- |
| **Demographic data** |  |  |  |  |
| Male [n (%)] | 1656 (57.9) | 1100 (57.7) | 556 (58.3) | 0.760 |
| Age (Mean ± SD, years) | 77.04 ± 7.84 | 77.31 ± 7.86 | 76.50 ± 7.79 | 0.052 |
| BMI (Mean ± SD) | 24.17 ± 2.94 | 24.22 ± 2.96 | 24.07 ± 2.90 | 0.190 |
| Waist circumference (Mean±SD, cm) | 83.35 ± 8.21 | 83.39 ± 7.93 | 83.27 ± 8.74 | 0.727 |
| OCSP [n (%)] |  |  |  | 0.848 |
| TACI [n (%)] | 291 (10.2) | 190 (10.0) | 101 (10.6) |  |
| PACI [n (%)] | 1733 (60.6) | 1166 (61.1) | 567 (59.4) |  |
| POCI [n (%)] | 711 (24.9) | 468 (24.5) | 243 (25.5) |  |
| LACI [n (%)] | 126 (4.4) | 83 (4.4) | 43 (4.5) |  |
| TOAST [n (%)] |  |  |  | 0.095 |
| large-artery atherosclerosis [n (%)] | 1130 (39.5) | 742 (38.9) | 388 (40.7) |  |
| Cardio embolism [n (%)] | 405 (14.1) | 288 (15.1) | 117 (12.3) |  |
| small-vessel occlusion [n (%)] | 1261 (44.1) | 839 (44.0) | 422 (44.2) |  |
| stroke of other determined etiology [n (%)] | 23 (0.8) | 11 (0.6) | 12 (1.3) |  |
| stroke of undetermined etiology [n (%)] | 42 (1.5) | 27 (1.4) | 15 (1.6) |  |
| Symptoms to time of arrival [M (25%, 75%), hours] | 7.0 (1.9, 28.4) | 6.5 (1.9, 27.8) | 7.7 (2.0, 29.9) | 0.178 |
| Smoking status [n (%)] |  |  |  | 0.593 |
| Never smoke [n (%)] | 2141 (74.8) | 1436 (75.3) | 705 (73.9) |  |
| Used to smoke [n (%)] | 142 (5.0) | 96 (5.0) | 46 (4.8) |  |
| Still smoke [n (%)] | 578 (20.2) | 375 (19.7) | 203 (21.3) |  |
| **Previous history [n (%)]** |  |  |  |  |
| Myocardial infarction [n (%)] | 17 (0.6) | 9 (0.5) | 8 (0.8) | 0.229 |
| Hypertensive diseases [n (%)] | 2206 (77.1) | 1486 (77.9) | 720 (75.5) | 0.141 |
| Diabetes [n (%)] | 870 (30.4) | 579 (30.4) | 291 (30.5) | 0.938 |
| Atrial fibrillation [n (%)] | 487 (17.0) | 339 (17.8) | 148 (15.5) | 0.129 |
| Lipid metabolism disorders [n (%)] | 16 (0.6) | 13 (0.7) | 3 (0.3) | 0.169 |
| Cerebral hemorrhage [n (%)] | 71 (2.5) | 47 (2.5) | 24 (2.5) | 0.934 |
| Dementia [n (%)] | 40 (1.4) | 30 (1.6) | 10 (1.0) | 0.260 |
| Psychiatric disorders [n (%)] | 13 (0.5) | 6 (0.3) | 7 (0.7) | 0.202 |
| Chronic obstructive Pulmonary disease [n (%)] | 65 (2.3) | 45 (2.4) | 20 (2.1) | 0.656 |
| Hemorrhagic disease [n (%)] | 45 (1.6) | 30 (1.6) | 15 (1.6) | 0.999 |
| Family history of stroke [n (%)] | 9 (0.3) | 5 (0.3) | 4 (0.4) | 0.724 |
| Heart valve replacement surgery [n (%)] | 4 (0.1) | 3 (0.2) | 1 (0.10) | 1.000 |
| **The severity of the disease** |  |  |  |  |
| Pre-morbidity mRS score [M (25%, 75%)] | 2 (1, 2) | 2 (1, 2) | 2 (1, 2) | 0.208 |
| NIHSS score within 24-hour at admission [M (25%, 75%)] | 2 (1, 7) | 2 (1, 7) | 2 (1, 6) | 0.339 |
| Dysphagia [n (%)] | 445 (15.6) | 287 (15.2) | 158 (16.8) | 0.264 |
| MAP (Mean ± SD, mmHg) | 104.32 ± 13.39 | 104.21 ± 13.43 | 104.56 ± 13.31 | 0.513 |
| Pulse (Mean ± SD, Times/minute) | 77.64 ± 15.11 | 77.74 ± 15.21 | 77.43 ± 14.91 | 0.610 |
| **Special treatment plan** |  |  |  |  |
| Alteplase intravenous thrombolysis [n (%)] | 405 (14.2) | 283 (14.8) | 158 (16.8) | 0.138 |
| Arterial catheter reperfusion [n (%)] | 22 (0.8) | 15 (0.8) | 7 (0.8) | 0.863 |
| Thrombectomy treatment [n (%)] | 39 (1.4) | 29 (1.6) | 10 (1.1) | 0.292 |
| **Laboratory indicators** |  |  |  |  |
| LDL [M (25%, 75%), mmol/L] | 2.55 (1.94, 3.21) | 2.56 (1.96, 3.24) | 2.50 (1.91, 3.16) | 0.233 |
| Hcy [M (25%, 75%), umol/L] | 15.5 (11.8, 21.0) | 15.7 (11.9, 21.1) | 15.2 (11.8, 20.7) | 0.362 |
| HbA1c (Mean±SD) | 6.61 ± 1.63 | 6.61 ± 1.65 | 6.61 ± 1.57 | 0.928 |
| ABG [M (25%, 75%), mmol/L] | 5.7 (5.0, 7.2) | 5.0 (5.0, 7.0) | 5.8 (5.1, 7.3) | 0.780 |
| SHR (Mean±SD) | 0.85 ± 0.21 | 0.85 ± 0.21 | 0.85 ± 0.21 | 0.838 |
| SCr [M (25%, 75%), umol/L] | 73.50 (62.10, 88.00) | 74.10 (62.30, 88.94) | 72.10 (61.78, 86.33) | 0.068 |
| BUN [M (25%, 75%), mmol/L] | 5.2 (4.3, 6.5) | 5.2 (4.3, 6.5) | 5.1 (4.3, 6.5) | 0.560 |
| UA (Mean ± SD, umol/L) | 326.61 ± 102.74 | 326.96 ± 101.97 | 325.92 ± 104.31 | 0.798 |
| INR (Mean±SD) | 0.96 ± 0.17 | 0.97 ± 0.17 | 0.96 ± 0.16 | 0.325 |
| **Prognostic index** |  |  |  |  |
| Length of hospitalization[M(25%, 75%), days] | 13 (10, 16) | 13 (10, 16) | 13 (10, 16) | 0.137 |
| Died in hospital [n (%)] | 61 (2.1) | 43 (2.3) | 18 (1.9) | 0.521 |
| Total hospitalization expenses [M (25%, 75%), Thousand yuan] | 15.0 (11.0, 20.1) | 15.1 (1.1, 2.1) | 15.0 (11.4, 20.2) | 0.255 |
| Total hospitalization drug expenses [M (25%, 75%), Thousand yuan] | 7.2 (4.5, 10.8) | 7.3 (4.5, 11.0) | 7.1 (4.6, 10.5) | 0.420 |
| NIHSS score at discharge [M (25%, 75%)] | 2 (1, 6) | 2 (1, 6) | 2 (1, 6) | 0.466 |
| mRS score at discharge [M (25%, 75%)] | 2 (1, 3) | 2 (1, 3) | 2 (1, 3) | 0.503 |

**Abbreviation:** BMI, body mass index; OCSP, oxfordshire community stroke project; TACI, total anterior circulation infarct; PACI, partial anterior circulation infarct; POCI, posterior circulation infarct; LACI, lacunar infarct; TOAST, trial of org 10172 in acute stroke treatment; mRS, modified rankin scale; NIHSS, national institute of health stroke scale; MAP, mean arterial pressure; LDL, low-density lipoprotein; Hcy, homocysteine; HbA1c, glycated hemoglobin; ABG, admission blood glucose; SHR, stress hyperglycemia ratio; SCr, serum creatinine; BUN, Blood Urea Nitrogen; UA, Uric Acid; INR, international normalized ratio.
