# Supplementary material for: Construction and evaluation of a nomogram model for predicting the risk of hospital-acquired pneumonia in elderly patients with acute ischemic stroke
Source: BMC Geriatr. 2025 May 14;25:340. doi: 10.1186/s12877-025-05936-3 (PMC12080133; doi:10.1186/s12877-025-05936-3)
Supplement: Supplementary file 6 — Supplementary Material 6 [file 12877_2025_5936_MOESM6_ESM.doc]

Supplementary Table 4 Compares the baseline characteristics of HAP and non-HAP groups in the training group

| Variables | Total  (n = 1907) | Non-HAP  (n=1447) | HAP  (n=460) | *P* |
| --- | --- | --- | --- | --- |
| **Demographic data** |  |  |  |  |
| Male [n (%)] | 1100 (57.7) | 861 (59.5) | 239 (52.0) | 0.004 |
| Age (Mean ± SD, years) | 77.31 ± 7.86 | 76.00 ± 7.55 | 81.40 ± 7.41 | <0.001 |
| BMI (Mean ± SD) | 24.22 ± 2.96 | 24.23 ± 3.00 | 24.19 ± 2.82 | 0.769 |
| Waist circumference (Mean ± SD, cm) | 83.39 ± 7.93 | 83.57 ± 7.81 | 82.85 ± 8.30 | 0.103 |
| OCSP [n (%)] |  |  |  | <0.001 |
| TACI [n (%)] | 190 (10.0) | 109 (7.5) | 81 (17.6) |  |
| PACI [n (%)] | 1166 (61.1) | 887 (61.3) | 279 (60.7) |  |
| POCI [n (%)] | 468 (24.5) | 372 (25.7) | 96 (20.9) |  |
| LACI [n (%)] | 83 (4.4) | 79 (5.5) | 4 (0.9) |  |
| TOAST [n (%)] |  |  |  | <0.001 |
| large-artery atherosclerosis [n (%)] | 742 (38.9) | 545 (37.7) | 197 (42.8) |  |
| Cardio embolism [n (%)] | 288 (15.1) | 166 (11.5) | 122 (26.5) |  |
| small-vessel occlusion [n (%)] | 839 (44.0) | 710 (49.1) | 129 (28.0) |  |
| stroke of other determined etiology [n (%)] | 11 (0.6) | 8 (0.6) | 3 (0.7) |  |
| stroke of undetermined etiology [n (%)] | 27 (1.4) | 18 (1.2) | 9 (2.0) |  |
| Symptoms to time of arrival [M (25%,75%), hours] | 6.5 (1.9, 27.8) | 7.6 (2.0, 30.4) | 4.6 (1.7, 18.9) | 0.007 |
| Smoking status [n (%)] |  |  |  | <0.001 |
| Never smoke [n (%)] | 1436 (75.3) | 1129 (78.0) | 307 (66.7) |  |
| Used to smoke [n (%)] | 96 (5.0) | 74 (5.1) | 22 (4.8) |  |
| Still smoke [n (%)] | 375 (19.7) | 244 (16.9) | 131 (28.5) |  |
| **Previous medical history [n (%)]** |  |  |  |  |
| Myocardial infarction [n (%)] | 9 (0.5) | 5 (0.3) | 4 (0.9) | 0.153 |
| Hypertensive diseases [n (%)] | 1486 (77.9) | 1121 (77.5) | 365 (79.3) | 0.398 |
| Diabetes [n (%)] | 579 (30.4) | 437 (30.2) | 142 (30.9) | 0.786 |
| Atrial fibrillation [n (%)] | 339 (17.8) | 205 (14.2) | 134 (29.1) | <0.001 |
| Lipid metabolism disorders [n (%)] | 13 (0.7) | 8 (0.6) | 5 (1.1) | 0.225 |
| Cerebral hemorrhage [n (%)] | 47 (2.5) | 32 (2.2) | 15 (3.3) | 0.206 |
| Dementia [n (%)] | 30 (1.6) | 21 (1.5) | 9 (2.0) | 0.448 |
| Psychiatric disorders [n (%)] | 6 (0.3) | 4 (0.3) | 2 (0.4) | 0.597 |
| Chronic obstructive Pulmonary disease [n (%)] | 45 (2.4) | 32 (2.2) | 13 (2.8) | 0.449 |
| Hemorrhagic disease [n (%)] | 30 (1.6) | 23 (1.6) | 7 (1.5) | 0.919 |
| Family history of stroke [n (%)] | 5 (0.3) | 2 (0.1) | 3 (0.7) | 0.06 |
| Heart valve replacement surgery [n (%)] | 3 (0.2) | 3 (0.2) | 0 (0) | 0.328 |
| **The severity of the disease** |  |  |  |  |
| premorbidity mRS score [M (25%, 75%)] | 2 (1, 2) | 2 (1, 2) | 2 (1, 2) | <0.001 |
| NIHSS score within 24 hours of admission [M (25%, 75%)] | 2 (1, 7) | 2 (1, 4) | 8 (3, 14) | <0.001 |
| Dysphagia [n (%)] | 287 (15.2) | 69 (4.8) | 218 (47.4) | <0.001 |
| MAP (Mean ± SD, mmHg) | 104.21 ± 13.43 | 104.39 ± 12.97 | 103.65 ± 14.79 | 0.304 |
| Pulse (Mean ± SD, Times/minute) | 77.74 ± 15.21 | 76.72 ± 14.07 | 80.95 ± 17.99 | <0.001 |
| **Special treatment plan** |  |  |  |  |
| Alteplase intravenous thrombolysis [n (%)] | 283 (14.8) | 206 (14.2) | 77 (16.7) | 0.188 |
| Arterial catheter reperfusion [n (%)] | 15 (0.8) | 11 (0.8) | 4 (0.9) | 0.843 |
| Thrombectomy treatment [n (%)] | 29 (1.6) | 18 (1.3) | 11 (2.5) | 0.089 |
| **Laboratory indicators** |  |  |  |  |
| LDL [M (25%,75%), mmol/L] | 2.56 (1.96, 3.24) | 2.59 (2.00, 3.25) | 2.48 (1.80, 3.15) | 0.066 |
| Hcy [M (25%,75%), umol/L] | 15.7 (11.9, 21.1) | 15.1 (11.7, 20.2) | 17.9 (12.7, 23.1) | <0.001 |
| HbA1c (Mean ± SD) | 6.61 ± 1.65 | 6.61 ± 1.62 | 6.64 ± 1.75 | 0.658 |
| ABG [M (25%,75%), mmol/L] | 5.0 (5.0, 7.0) | 5.6 (5.0, 7.0) | 6.2 (5.3, 7.8) | <0.001 |
| SHR (Mean ± SD) | 0.85 ± 0.21 | 0.82 ± 0.18 | 0.93 ± 0.27 | <0.001 |
| SCr [M (25%,75%), umol/L] | 74.10 (62.30, 88.94) | 73.50 (62.15, 87.66) | 76.10 (62.50, 93.28) | <0.001 |
| BUN [M (25%,75%), mmol/L] | 5.2 (4.3, 6.5) | 5.1 (4.3, 6.2) | 5.6 (4.5, 7.5) | <0.001 |
| UA (Mean ± SD, umol/L) | 326.96 ± 101.97 | 325.88 ± 95.15 | 330.34 ± 121.00 | 0.415 |
| INR (Mean ± SD) | 0.97 ± 0.17 | 0.96 ± 0.16 | 1.01 ± 0.19 | <0.001 |
| **Prognostic index** |  |  |  |  |
| Length of hospitalization[M(25%,75%), days] | 13 (10, 16) | 12 (10, 15) | 17 (12, 22) | <0.001 |
| Died in hospital [n (%)] | 43 (2.3) | 8 (0.6) | 35 (7.6) | <0.001 |
| Total hospitalization expenses [M (25%,75%), Thousand yuan] | 15.1 (1.1, 2.1) | 14.3 (11.0, 18.6) | 21.8 (14.9, 33.4) | <0.001 |
| Total hospitalization drug expenses [M (25%,75%), Thousand yuan] | 7.3 (4.5, 11.0) | 6.8 (4.2, 9.9) | 10.8 (6.4, 16.0) | <0.001 |
| NIHSS score at discharge [M (25%,75%)] | 2 (1, 6) | 2 (1, 4) | 8 (2, 14) | <0.001 |
| mRS score at discharge [M (25%,75%)] | 2 (1, 3) | 1 (1, 3) | 4 (2, 5) | <0.001 |

**Abbreviation:** HAP, hospital-acquired pneumonia; BMI, body mass index; OCSP, oxfordshire community stroke project; TACI, total anterior circulation infarct; PACI, partial anterior circulation infarct; POCI, posterior circulation infarct; LACI, lacunar infarct; TOAST, trial of org 10172 in acute stroke treatment; mRS, modified rankin scale; NIHSS, national institute of health stroke scale; MAP, mean arterial pressure; LDL, low-density lipoprotein; Hcy, homocysteine; HbA1c, glycated hemoglobin; ABG, admission blood glucose, SHR, stress hyperglycemia ratio; SCr, serum creatinine; BUN, Blood Urea Nitrogen; UA, Uric Acid; INR, international normalized ratio.
